# Quantitative analysis of the interplay between hsc70 and its co-chaperone HspBP1

**DOI:** 10.7717/peerj.1530

**Published:** 2015-12-21

**Authors:** Hicham Mahboubi, Ursula Stochaj

**Affiliations:** Department of Physiology, McGill University, Montreal, Quebec, Canada

**Keywords:** Chaperone, Co-chaperone, Heat shock protein, Stress response, Single-cell analysis, Subcellular protein distribution, Quantitative microscopy, Regression analysis

## Abstract

**Background.** Chaperones and their co-factors are components of a cellular network; they collaborate to maintain proteostasis under normal and harmful conditions. In particular, hsp70 family members and their co-chaperones are essential to repair damaged proteins. Co-chaperones are present in different subcellular compartments, where they modulate chaperone activities.

**Methods and Results.** Our studies assessed the relationship between hsc70 and its co-factor HspBP1 in human cancer cells. HspBP1 promotes nucleotide exchange on hsc70, but has also chaperone-independent functions. We characterized the interplay between hsc70 and HspBP1 by quantitative confocal microscopy combined with automated image analyses and statistical evaluation. Stress and the recovery from insult changed significantly the subcellular distribution of hsc70, but had little effect on HspBP1. Single-cell measurements and regression analysis revealed that the links between the chaperone and its co-factor relied on (i) the physiological state of the cell and (ii) the subcellular compartment. As such, we identified a linear relationship and strong correlation between hsc70 and HspBP1 distribution in control and heat-shocked cells; this correlation changed in a compartment-specific fashion during the recovery from stress. Furthermore, we uncovered significant stress-induced changes in the colocalization between hsc70 and HspBP1 in the nucleus and cytoplasm.

**Discussion.** Our quantitative approach defined novel properties of the co-chaperone HspBP1 as they relate to its interplay with hsc70. We propose that changes in cell physiology promote chaperone redistribution and thereby stimulate chaperone-independent functions of HspBP1.

## Introduction

The prevention of and recovery from stress-induced injuries require factors that repair cellular damage. Heat shock proteins, in particular members of the hsp70 family and their co-factors, are essential for these repair processes (Bracher & Verghese, 2015; Hartl, Bracher & Hayer-Hartl, 2011). A complex network of chaperones and co-chaperones collaborates to promote cell survival in response to diverse types of injury and stress (Brandvold & Morimoto, 2015; Gyurko et al., 2014; Palotai, Szalay & Csermely, 2008; Pratt et al., 2015; Richter, Haslbeck & Buchner, 2010). Furthermore, regulated chaperone and co-chaperone activities are critical to prevent the loss of proteostasis, a hallmark of many human pathologies, ranging from cancer, diabetes and aging to neurodegenerative disorders (Csermely, 2001; Kakkar et al., 2014; Palotai, Szalay & Csermely, 2008; Scheibel & Buchner, 2006; Wang, Facciponte & Subjeck, 2006).

One well-established method to alter protein homeostasis is acute hyperthermia which initiates the heat shock response. A key step of this response is the activation of heat shock factor 1 (Hsf-1) in the cytoplasm. This activation leads to Hsf-1 accumulation in the nucleus, where it binds heat shock elements that are present in the promoters of many chaperone genes. The subsequent upregulation of gene expression is accompanied by post-transcriptional and translational changes. Together, these events culminate in the elevated production of chaperones that are necessary to repair damaged proteins and restore proteostasis (reviewed in Morimoto, 2011; Sonna et al., 2002; Vihervaara & Sistonen, 2014). Notably, not all heat-inducible chaperone gene promoters contain consensus heat shock elements (Finka, Mattoo & Goloubinoff, 2011), indicating that additional mechanisms stimulate their transcription upon hyperthermia.

Chaperones have compartment-specific functions; depending on their subcellular localization, they can contribute to different biological processes (Banski, Kodiha & Stochaj, 2010; Banski et al., 2010; Escusa-Toret, Vonk & Frydman, 2013; Hageman et al., 2007; Stolz & Wolf, 2010; Young, Barral & Ulrich Hartl, 2003). These compartment-associated activities rely on the presence of co-chaperones, as exemplified by HspBP1. While co-chaperones are critical regulators of chaperone cycles, they can also provide biological activities that do not require chaperones (Shiber & Ravid, 2014).

HspBP1 interacts with hsc70 and other members of the hsp70 family (Kabani et al., 2002; Taipale et al., 2014); the co-chaperone was proposed to function as a nucleotide exchange factor for hsp70/hsc70. HspBP1 concentrations increase in some forms of cancer (Graner et al., 2009; Souza et al., 2009; Yang et al., 2015), suggesting a possible role in tumor biology. For instance, it is conceivable that the coordinated interaction between HspBP1 and hsp70 family members regulates tumor cell survival (Graner et al., 2009; Tanimura et al., 2007). Interestingly, several anticancer drugs target HspBP1 and modulate the activity of its hsp/hsc70 binding partners and their pro-survival function (Tanimura et al., 2007).

Aside from cancer, HspBP1 participates in other processes that are relevant to proteostasis and cell signaling. For example, HspBP1 inhibits CHIP-mediated degradation of CFTR and thereby enhances the production of mature channels (Alberti et al., 2004). Furthermore, HspBP1 reduces hsc70 binding to steroid hormone receptors (Knapp et al., 2014) and regulates spermatogenesis by inhibiting the degradation of two inducible hsp70 family members (Rogon et al., 2014).

Proteomics studies examined the subcellular content (Boisvert et al., 2012, reviewed by Kodiha, Frohlich & Stochaj, 2012) or copy number (Finka et al., 2015) of chaperones and co-chaperones. In the absence of stress, hsc70 and HspBP1 are mostly present in cytoplasmic fractions of HeLa cells (Boisvert et al., 2012). Interestingly, mild heat stress increases hsc70 copy numbers/cell in Jurkat T cells (Finka et al., 2015).

While proteomics can generate large data sets for the abundance and modification of chaperones and their co-factors, spatial proteomics depends on reliable cell fractionation. This method is especially error-prone for the nucleus and its subcompartments, because proteins often “leak” out of the nucleus during isolation (Liu & Fagotto, 2011). Using fixed cells as starting material is therefore a valuable method to gain spatial information on chaperones and their co-factors. In combination with appropriate image acquisition and analysis, this spatial information will be quantitative (Kodiha, Banski & Stochaj, 2011; Kodiha, Brown & Stochaj, 2008; Kodiha et al., 2015). We have applied these protocols earlier to measure the nucleolar accumulation of hsc70 in heat-stressed cells (Banski et al., 2010). At present, comprehensive imaging data are not available for HspBP1. However, they are required to define the spatial distribution of HspBP1, especially in relation to its binding partners, such as the chaperone hsc70.

To gain such insights, we conducted a series of quantitative studies that evaluated the interplay between HspBP1 and hsc70. We define interplay as the “reciprocal relationship” between both proteins; it includes, but is not limited to, the colocalization of hsc70 and HspBP1. Specifically, our experiments measure the subcellular distribution and abundance of endogenous HspBP1 and its chaperone partner hsc70, a constitutively synthesized hsp70 family member. This work was performed in human cancer cells under normal growth conditions, upon heat shock and during the recovery from stress. Our study provides data at the single-cell level; we compare the steady-state distribution and the compartment-specific interplay between the chaperone/co-chaperone pair hsc70/HspBP1. In addition to these assessments, we quantified the colocalization of hsc70 and HspBP1 in the nucleus and cytoplasm during and after acute stress.

Taken together, our experiments and statistical analyses reveal a complex relationship between the chaperone hsc70 and its co-chaperone HspBP1 inside the cell. We uncover at the single-cell level specific changes that occur in different cellular compartments during stress exposure and upon recovery. Thus, our data shed new light on the coordinated response to stress as it relates to the hsc70/HspBP1 pair. We propose that these events are important to maintain cellular proteostasis under stress and disease conditions.

## Materials and Methods

### Cell culture and heat shock

HeLa cells were grown in Dulbecco’s Modified Eagle Medium (DMEM) supplemented with antibiotics and 8% Fetal Bovine Serum (FBS). Cells were maintained at 37 °C; they were grown to 70% confluency on poly-lysine-coated cover slips and analyzed between passages 3 and 12. For heat shock, cells were exposed to 45.5 °C for 1 h; subsequent recovery was at 37 °C for different periods of time.

### Immunocytochemistry

HeLa cells were washed with PBS, fixed for 10 min with 10% formaldehyde in PBS, permeabilized for 5 min with cold acetone at −20 °C and blocked for 1 h with PBS/ 2 mg/ml BSA/1 mM NaN_3_ (blocking solution). All subsequent steps were carried out in blocking solution at room temperature. Samples were incubated for 1 h with primary antibodies against hsc70 (diluted 1:1,000; Stressgen SPA-815), HspBP1 (diluted 1:200; Santa Cruz sc-34252) or Crm1 (diluted 1:200, sc-5595). Following three washes, fluorescently-labeled secondary antibodies were added for 1 h. Samples were washed and nuclei stained for 2 min with 1 µg/ml 4,6′-diamidino-2-phenylindole (DAPI). Cover slips were mounted and sealed.

### Microscopy and quantitative image analysis

Images were acquired in the multi-track mode with a Zeiss LSM510 confocal microscope. Appropriate filter settings were selected to minimize cross-talk between the channels.

Image quantification was performed with MetaXpress software, as described earlier (Kodiha, Brown & Stochaj, 2008, Fig. S1). In brief, the DAPI signal defined the nuclear and nucleolar compartments. Crm1, another nuclear marker, validated the use of DAPI to demarcate nuclei. The first step of image analysis was background correction (Kodiha, Brown & Stochaj, 2008). To this end, average pixel intensities from a region devoid of cells were subtracted to generate a *Background Correction image*. After background correction, pixel intensities of the proteins of interest were measured in the nucleus and cytoplasm. All steps were automated. At least 35 cells were quantified for each of the different conditions in every experiment. All images were visually inspected to verify the correctness of the compartment demarcation. For single-cell analysis, a minimum of 115 cells was quantified for each of the five time points. For each experiment, data were normalized to non-stressed controls.

Colocalization was measured with MetaXpress software as follows: regions of interest (ROI) that were identical for hsc70 and HspBP1 channels were selected for the nucleus or cytoplasm. For each cell analyzed, the colocalization was quantified both in the nucleus and cytoplasm (Fig. S2). The images were thresholded, and the overlap between hsc70 and HspBP1 signals was evaluated for the same pixel using the Colocalization Module. The pixel size was 0.14 µm^2^. The same threshold value for each channel was used for all cells and maintained across the different conditions. At least 10 cells were analyzed per condition. The results show percent overlap of the hsc70 with HspBP1 area (Hsc70/HspBP1), or percent overlap of the HspBP1 with hsc70 area (HspBP1/hsc70). Different steps of the method and representative output data are shown in Fig. S2.

### Western blotting

Methods for the preparation of whole cell extracts and Western blotting have been published (Mahboubi, Barisé & Stochaj, 2015). Primary antibodies were used at the following dilutions: hsc70 (1:10,000), HspBP1 (1:400), and actin (1:100,000; Chemicon; EMD Millipore, Hayward, CA, USA).

### Statistics

Data were obtained for at least three independent experiments for microscopy and Western blot analyses. Significant differences (*p* < 0.05) were identified with One-way ANOVA and Bonferroni post-hoc analysis. Results are shown as means + SEM. Linear regression analysis was performed in Microsoft Excel; the equations and *R*^2^ values are included in the scatter plots. The Pearson correlation coefficient was computed using the standard formula *r* = cov (hsc70, HspBP1)/[*σ*_Hsc70_ × *σ*_HspBP1_].

## Results and Discussion

### Rationale for the quantitative analyses of the co-chaperone HspBP1 and its binding partner hsc70

The objective of this study was to define the reciprocal relationship between HspBP1 and hsc70 and gain quantitative spatial information on their possible interplay under normal and stress conditions. Our work focused on hsc70, rather than other HspBP1 interactors, for several reasons. The BioGrid database (Chatr-Aryamontri et al., 2015) lists hsc70 (also referred to as HspA8) as the most frequently identified binding partner for HspBP1. Hsc70 is essential for mammalian cell survival (Daugaard, Rohde & Jäättelä, 2007 and references therein). Moreover, primate hsc70, but not inducible hsp70, supports growth of the budding yeast *Saccharomyces cerevisiae* (Tutar, Song & Masison, 2006), emphasizing the important and conserved role of hsc70. The chaperone is constitutively synthesized; it is thus available to interact with HspBP1 in the absence and presence of stress. In the human brain, aging and neurodegeneration, conditions associated with chronic stress, reduce the expression of HspBP1 and hsc70 genes, while hsp70 gene expression is upregulated (Brehme et al., 2014). This may indicate that HspBP1 and hsc70 act in a coordinated fashion, at least during chronic stress. Based on their importance for basic biological processes (see ‘Introduction’) and human disease, hsc70 and HspBP1 as well as their interplay are of particular interest to human physiology.

**Figure 1:**
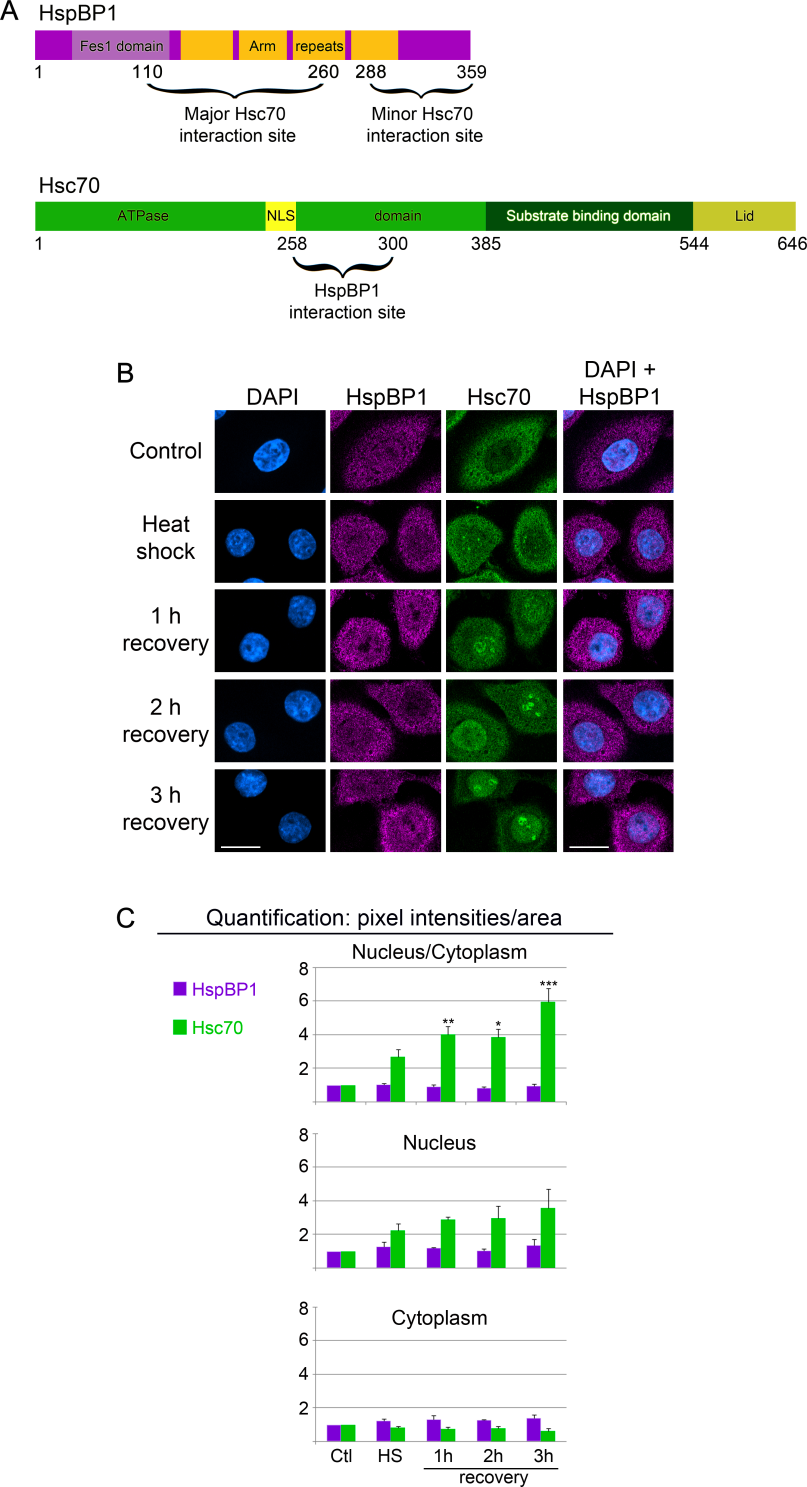
Subcellular distribution of the co-chaperone HspBP1 and its binding partner hsc70 under control and stress conditions. (A) HspBP1 and hsc70 organization. Domains are depicted for HspBP1 and hsc70, numbers denote amino acid residues. The regions involved in the co-chaperone/chaperone interactions are marked; they are based on the crystal structure (Shomura et al., 2005). NLS denotes the nucleolar localization sequence (Banski et al., 2010). Hsc70 and HspBP1can be modified posttranslationally; some of these modifications occur in the chaperone/co-chaperone interaction sites (Table 1, (Hornbeck et al., 2015)). (B) HeLa cells were grown at 37 °C or exposed to heat shock, followed by stress recovery. Representative confocal images for the immunolocalizations of HspBP1 and hsc70 are shown. Nuclei were detected with DAPI; an overlay of DAPI and HspBP1 images demarcates the nuclear compartments. Size bars are 20 µm. (C) Pixel intensities were quantified in the nucleus or cytoplasm, and the nucleocytoplasmic ratio was calculated. Results are shown as means + SEM for three independent experiments. Significant differences were identified by One-Way ANOVA, using unstressed control cells as the reference; ^∗^*p* < 0.05, ^∗∗^*p* < 0.01, ^∗∗∗^*p* < 0.001.

Our earlier work provided initial studies of the nuclear and nucleolar association of hsc70 (Banski et al., 2010; Kodiha et al., 2005), whereas little is known about the subcellular distribution of HspBP1. As previously, the current studies use HeLa cells as an established model system to examine heat shock proteins and their subcellular location (Banski et al., 2010; Kodiha et al., 2005). Figure 1A shows the domain organization of HspBP1 and hsc70 and the regions that participate in the interaction between both proteins.

### Assessment of the subcellular distribution of hsc70 and its co-chaperone HspBP1 during different growth conditions

To define the impact of a changing environment on the chaperone/co-chaperone pair hsc70/HspBP1, we evaluated endogenous proteins by quantitative immunofluorescence. This method was selected to avoid the complications associated with cell fractionation (Kodiha et al., 2014 and references therein). The localization of hsc70 and HspBP1 was monitored in non-stressed cells, during heat shock and at different time points of post-stress recovery (1, 2 and 3 h). Hsc70 and HspBP1 localized to the nucleus and cytoplasm in unstressed HeLa cells (Fig. 1B), and the exposure to heat shock induced a robust accumulation of hsc70 in the nucleus. During the recovery period, hsc70 began to concentrate in nucleoli (Fig. 1B and Banski et al., 2010), which were identified as “dark holes” in the DAPI image (Fig. S1; Kodiha, Banski & Stochaj, 2011). Under the same stress conditions, there was little effect on the overall distribution of HspBP1 (Fig. 1B). Notably, HspBP1 remained largely excluded from nucleoli. In summary, stress markedly impinged on the steady-state distribution of hsc70, but only minor changes were observed for HspBP1.

### Quantification of the compartment-specific changes in hsc70 and HspBP1 localization

**Figure 2:**
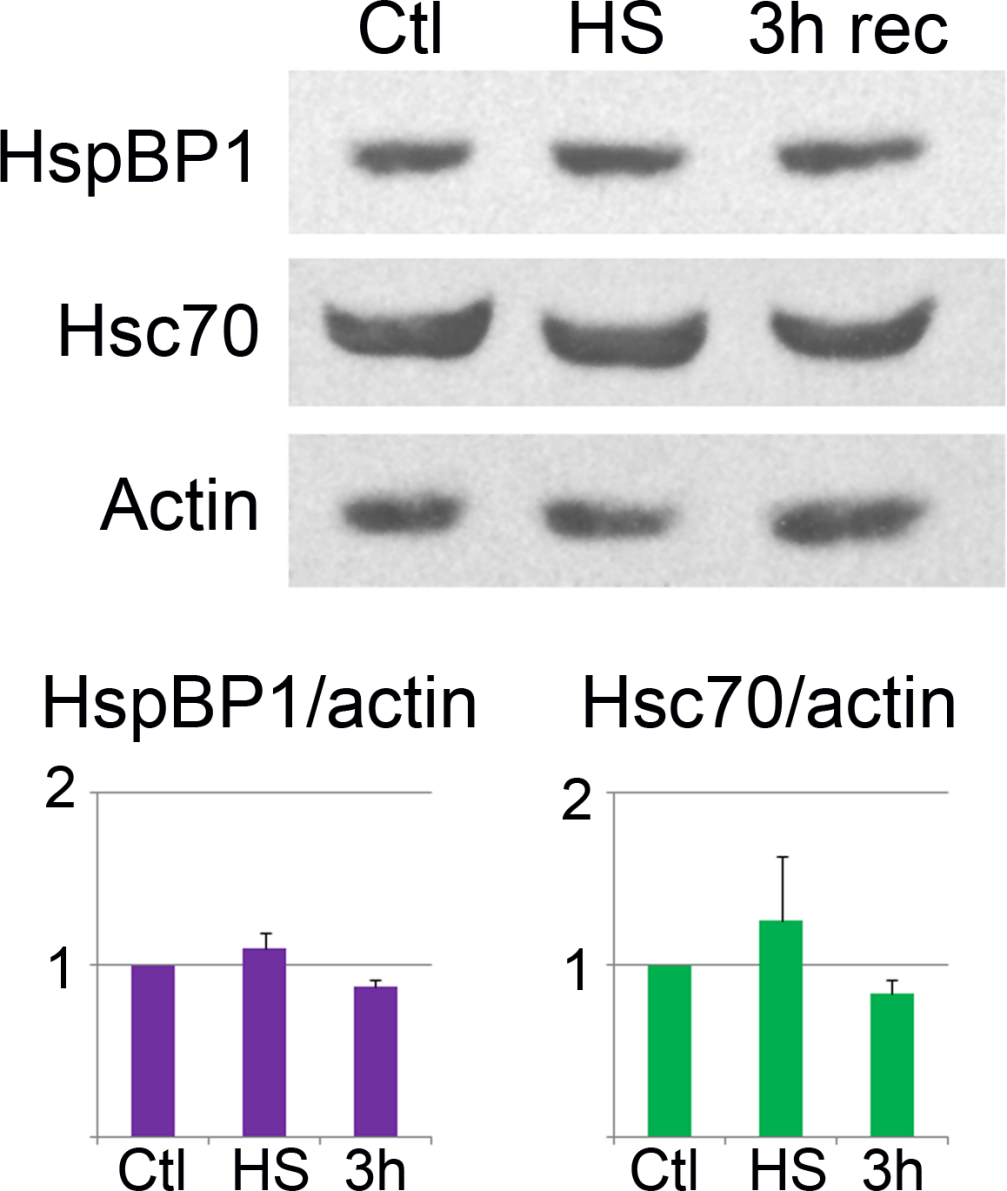
Western blot analysis of hsc70 and HspBP1. HeLa cells were grown under non-stress control conditions (Ctl) or exposed to heat shock (HS), followed by 3 h recovery (3 h rec). Crude extracts were examined by Western blotting with antibodies against HspBP1, hsc70 or actin. Enhanced chemiluminescence signals were quantified and normalized to actin. The graph shows means + SEM for three independent experiments. There were no significant changes for the abundance of HspBP1 or hsc70.

The proper assessment of protein redistribution can be complicated by changes in protein abundance, such as *de novo* synthesis or degradation. We therefore compared the cellular concentrations of hsc70 and HspBP1 in control, heat-shocked and recovering cells. To this end, Western blotting was performed on whole-cell extracts (Fig. 2), and protein levels were quantified for the time points relevant to our microscopic studies. As compared to control cells, hsc70 and HspBP1 levels did not change significantly throughout the stress and recovery period. This is consistent with results we reported earlier for hsc70 (Banski et al., 2010). These properties distinguish hsc70 and HspBP1 from other components of the chaperone network, because many chaperone and co-chaperone concentrations increase upon stress (Hageman et al., 2007 and references therein).

**Table 1:** Posttranslational modifications in hsc70/HspBP1 interaction sites. PhosphoSitePlus (Hornbeck et al., 2015) reports more than 130 modifications for human hsc70 and 12 for human HspBP1. Table 1 lists only modifications that are located in hsc70/HspBP1 binding sites; they include phosphorylation (p) and ubiquitination (ub). Note that PhosphoSitePlus numbering is for the HspBP1 isoform that contains 362, rather than 359 amino acid residues as depicted in Fig. 1 and used to solve the crystal structure (Shomura et al., 2005). The 362 amino acid isoform contains three additional glycine residues inserted after residue 30 of the 359 amino acid isoform.

Human hsc70		Human HspBP1	
T265-p	RAVRRLRtACERAKR	T206-p	LDRDACDtVRVkALF
T273-p	ACERAKRtLSSsTQA	K210-ub	ACDtVRVkALFAISC
S277-p	AKRtLSSsTQASIEI	S236-p	FLRLDGFsVLMRAMQ
		K248-ub	AMQQQVQkLkVKSAF
		K250-ub	QQQVQkLkVKSAFLL
		S353-p	KLLQTCFssPADDsM
		S354-p	LLQTCFssPADDsMD
		S359-p	FssPADDsMDR

To determine whether hsc70 and HspBP1 share additional properties, their subcellular location was measured with quantitative microscopy methods that we established previously (Kodiha, Brown & Stochaj, 2008; Kodiha, Mahboubi & Stochaj, 2014). As shown in Fig. 1C, the distribution of hsc70 changed in response to stress, as cells displayed a time-dependent increase in nuclear fluorescence. At the same time, cytoplasmic signals were reduced. (It should be noted that the figure shows pixel intensities/area. Therefore, the gain in nuclear fluorescence is not identical to the loss of cytoplasmic signals). Changes in hsc70 distribution were especially pronounced during the recovery period, leading to a significant increase of the nucleus/cytoplasm ratio (Fig. 1C). Interestingly, this global analysis uncovered only minor effects for HspBP1, because none of the conditions caused a significant relocation of the co-chaperone.

Taken together, our new data demonstrate significant differences in the subcellular distribution of hsc70 and HspBP1 upon stress. Under these conditions, there was no strong link between the steady-state distribution of hsc70 and its co-chaperone HspBP1. This could suggest that during heat shock and stress recovery hsc70 and HspBP1 contribute –at least in part- to different functions. Our hypothesis is supported by the growing list of HspBP1 non-chaperone binding partners that participate in cytoplasmic and nuclear functions (Chatr-Aryamontri et al., 2015).

### Single-cell analyses for HspBP1 and hsc70

Results presented in Fig. 1 demonstrate significant global changes for the subcellular hsc70 distribution, with only small changes for HspBP1. However, the experiments above do not provide information on whether these changes affect all or only a subpopulation of cells. To address this point, we went beyond the analyses in Fig. 1, and data were examined at the single-cell level.

**Figure 3:**
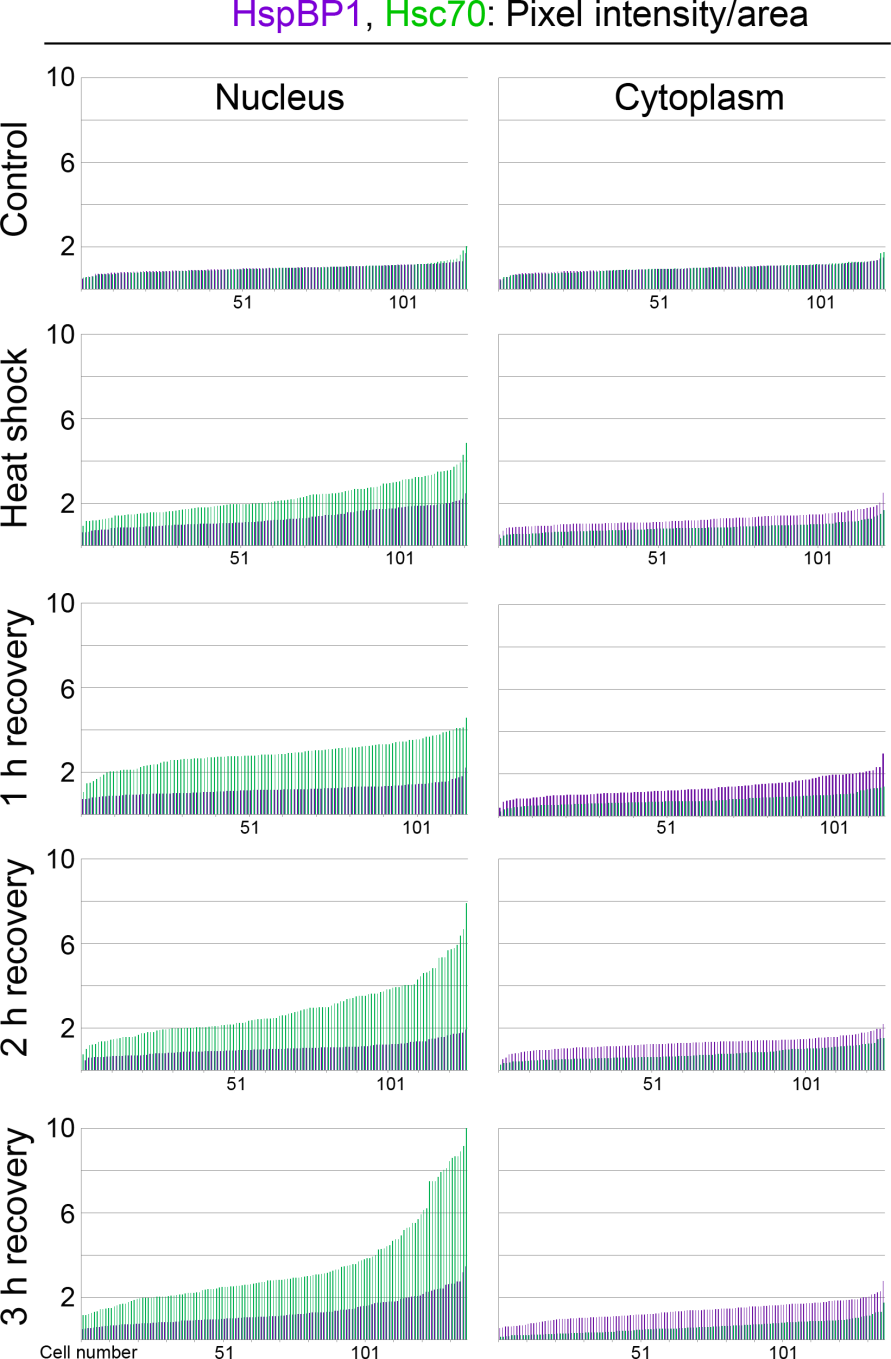
Single-cell analysis reveals subcellular changes in the pixel intensity/area for HspBP1 (purple) and hsc70 (green) in individual cells. Results are shown for three independent experiments. For each data set, pixel intensities were normalized to the average of the non-stress control sample. Data are plotted in ascending order. A minimum of 115 cells were examined for each condition.

Hsc70 and HspBP1 were evaluated in at least 115 cells for each time point. Pixel intensities were quantified in the nucleus and cytoplasm for hsc70 and HspBP1 in individual cells, and results were normalized to non-stress controls. In Fig. 3, data were plotted in ascending order for each individual nucleus and cytoplasm. They show that during hyperthermia and stress recovery the relative abundance of HspBP1 (purple) and hsc70 (green) shifted for the whole cell population. This applied to the nucleus, where a global increase in hsc70 abundance was detected. Interestingly, we uncovered that HspBP1 levels in the cytoplasm rise, while they diminish for hsc70.

**Figure 4:**
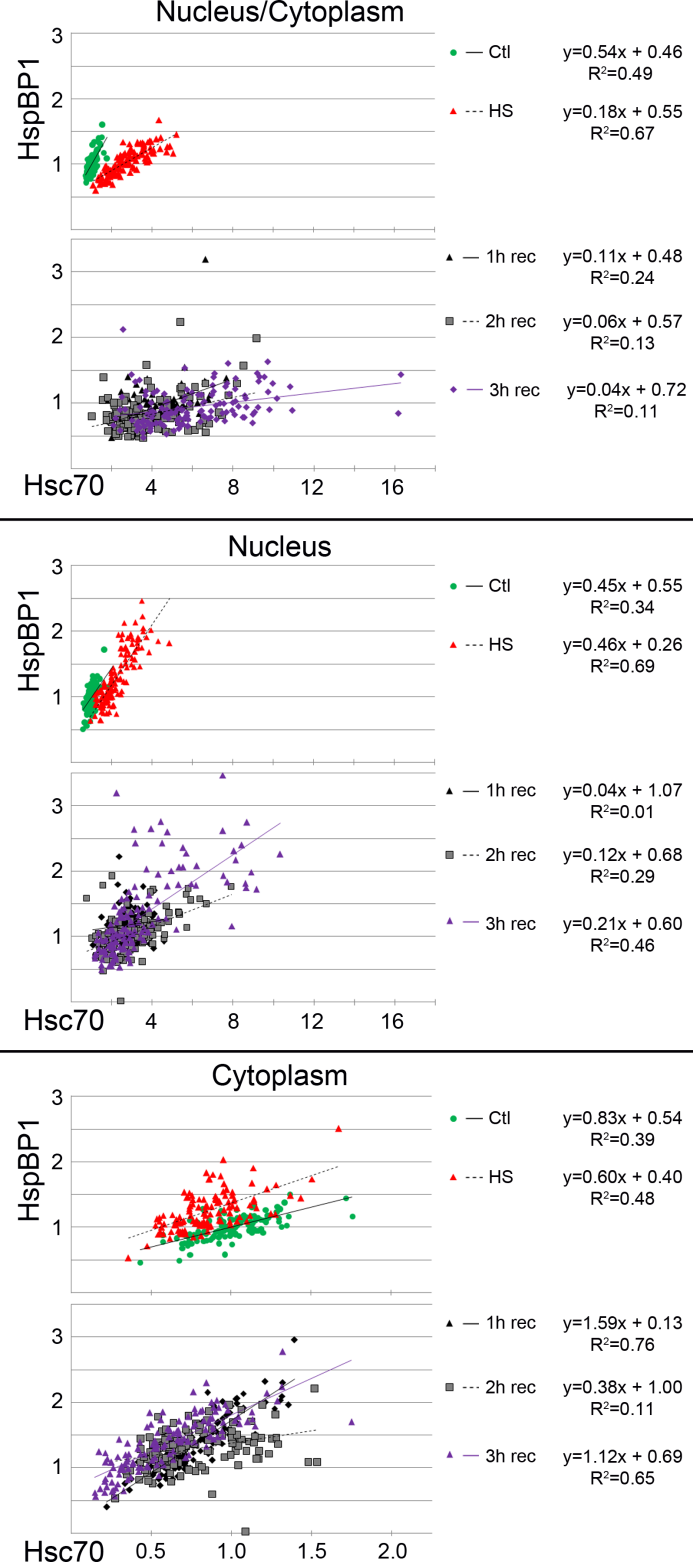
Regression analysis examines the interplay between hsc70 and HspBP1 at the single cell level. Quantitative image analysis was performed for control, heat-shocked and recovering cultures. Hsc70/HspBP1 data sets were obtained for the nucleus and cytoplasm of individual cells. Graphs depict single-cell results for three independent experiments. At least 115 cells were examined for each condition. Linear regression trendlines are included in the plots; functions and *R*^2^ values for the best fit are shown at the right margin.

To identify a possible link between HspBP1 and hsc70 abundance in *individual cells*, we performed regression analyses. Figure 4 plots single-cell fluorescence intensities for HspBP1 as a function of hsc70 signals. Plots were generated for (i) the ratio nucleus/cytoplasm, (ii) pixel intensities in the nucleus, and (iii) pixel intensities in the cytoplasm. Figure 4 suggests a linear relationship between the hsc70 and HspBP1 levels, both in the nucleus and cytoplasm under control and heat shock conditions. Interestingly, the slope of the regression line changed upon heat shock and during recovery, and the distribution of single-cell data were much more scattered in recovering cells (Fig. 4, see graphs for 1, 2, 3 h recovery). A likely interpretation of our work is that heat shock and stress recovery alter the interplay between this chaperone/co-chaperone pair.

**Figure 5:**
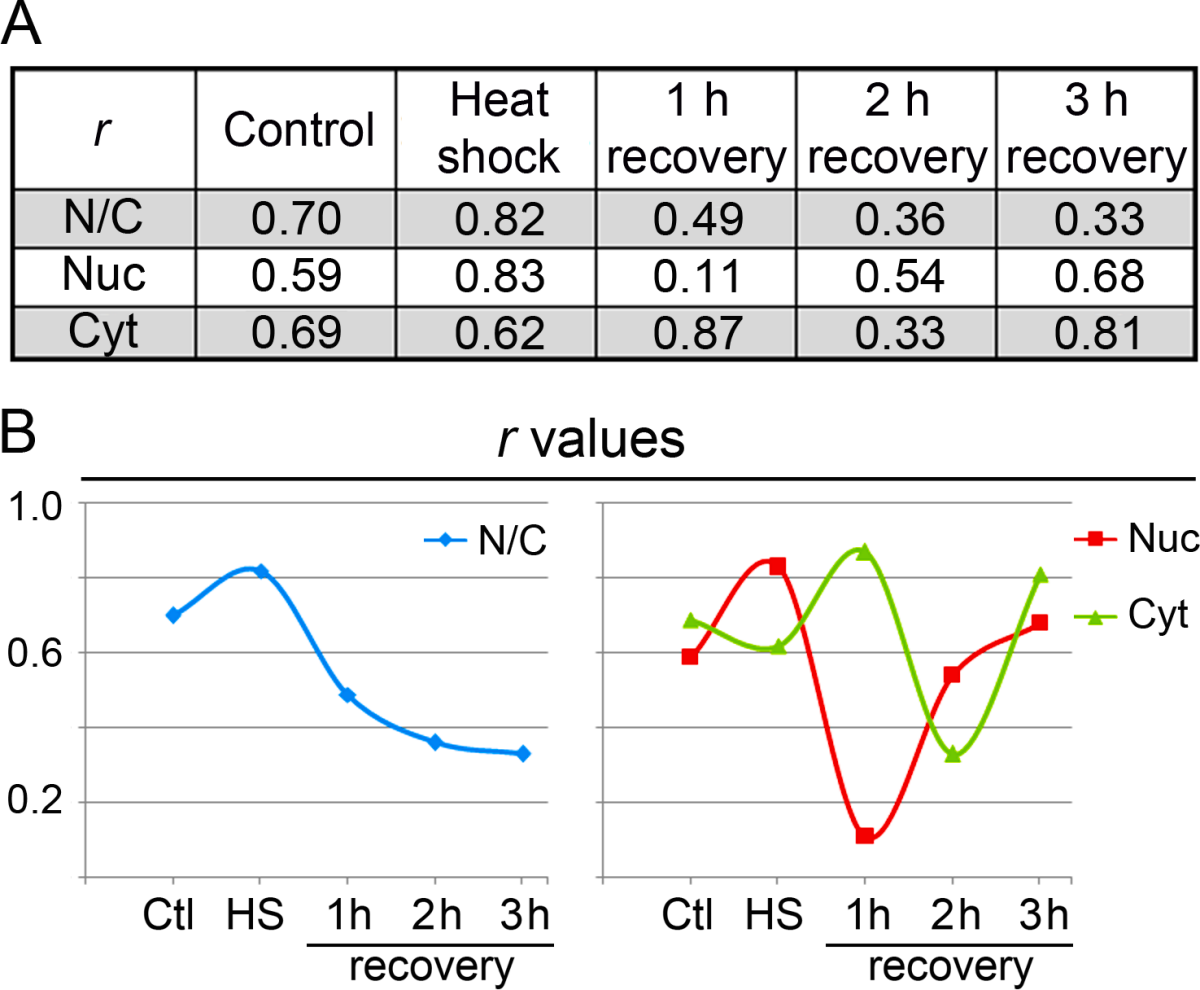
Pearson’s correlation coefficients for the hsc70/HspBP1 distribution. (A) The table depicts *r* values calculated for the pixel intensities of the hsc70/HspBP1 pair. Results are shown for the ratio nucleus/cytoplasm (N/C), the nucleus (Nuc) and cytoplasm (Cyt). (B) Graphical representation of *r* values for different experimental conditions.

To obtain a better understanding of this relationship, we computed the Pearson’s correlation coefficient (*r*) for single-cell data sets (Figs. 5A and 5B). Heat shock substantially increased the correlation coefficient in the nucleus. During recovery, there was a strong correlation between hsc70 and HspBP1 abundance in the cytoplasm at 1 and 3 h (*r* > 0.8). Interestingly, we observed the lowest *r* value in the nucleus at 1 h recovery.

Taken together, quantitative single-cell analyses showed that the stress-induced hsc70 relocation occurs in the whole cell population. Moreover, we uncovered small shifts for HspBP1 abundance, especially in the cytoplasm during stress recovery. On the single-cell level, the relationship between hsc70 and HspBP1 can be described by a linear function for control and heat shock conditions.

Overall, our results support the idea that the hsc70/HspBP1 interplay is (i) stress dependent, (ii) different in the cytoplasm and nucleus and (iii) affects the entire cell population.

### Nucleolar association of hsc70 and HspBP1 in control and stressed cells

**Figure 6:**
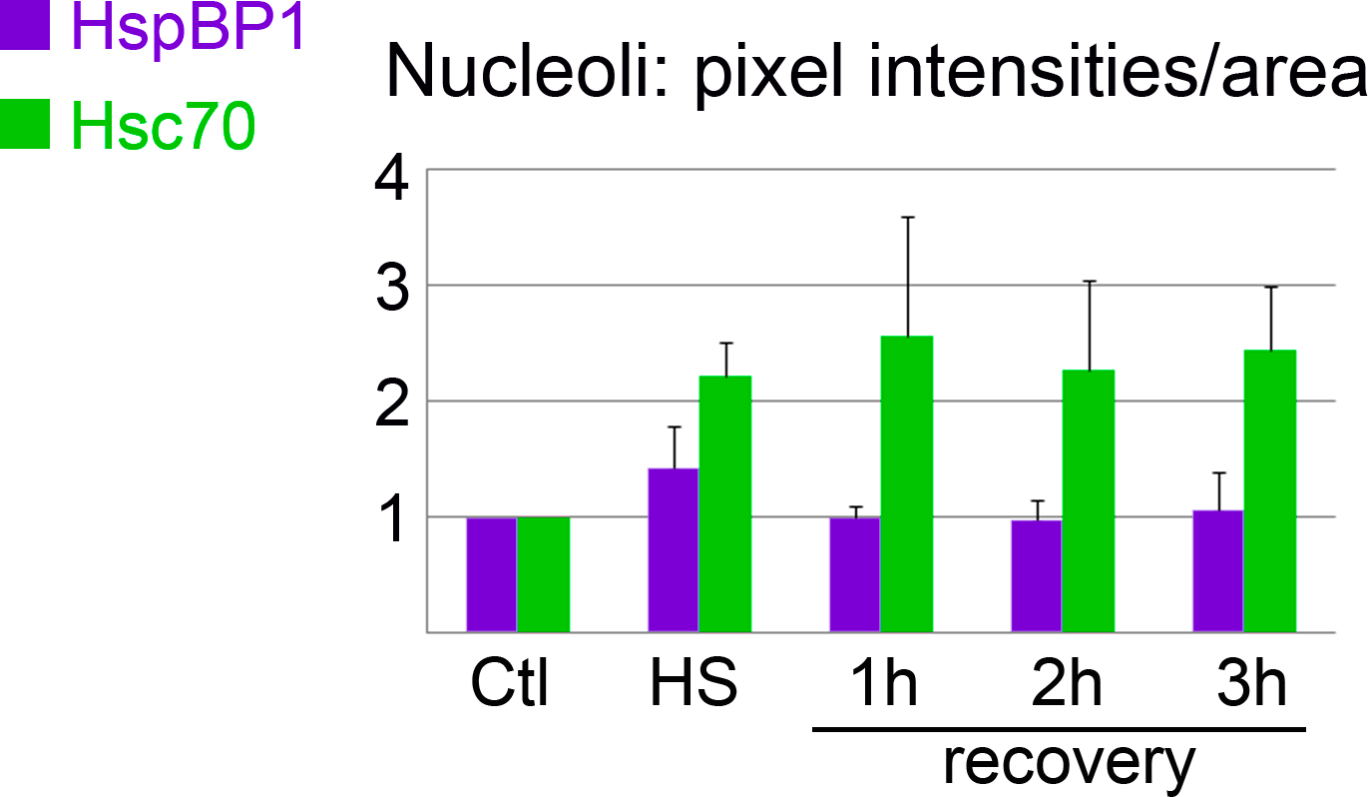
Nucleolar association of HspBP1 and hsc70 under control, heat shock and recovery conditions. Using previously published methods (Kodiha, Banski & Stochaj, 2011), pixel intensities in the nucleolar compartment were quantified for control or heat shock conditions and during stress recovery. Means + SEM are shown for three independent experiments.

Our earlier work (Banski et al., 2010; Kodiha et al., 2005) and Fig. 1 showed that hsc70 concentrated in nucleoli in a stress-dependent fashion. It was thus important to determine whether HspBP1 locates to nucleoli as well. This idea was tested with methods we developed previously (Kodiha, Banski & Stochaj, 2011). To this end, we applied protocols to automatically demarcate nucleoli (Fig. S1) and quantify signals within the nucleolar compartment. The measurements of nucleolar signals revealed hsc70 nucleolar accumulation during the recovery period (Figs. 1 and 6 and Banski et al., 2010). Unlike its binding partner hsc70, HspBP1 did not concentrate markedly in nucleoli at any of the examined time points (Figs. 1 and 6).

For the experiments presented here, the most pronounced hsc70 nucleolar accumulation occurred at 1 h recovery. Interestingly, this coincided with the lowest Pearson’s correlation coefficient (*r* = 0.11) for nuclear hsc70/HspBP1 data sets, as calculated for single-cell data (Fig. 5). The non-homogeneous hsc70 distribution in nuclei, due to nucleolar accumulation, may contribute to the low *r* value.

These new findings for HspBP1 are significant for the understanding of its co-chaperone function. Several studies support the idea that hsp70 family members play a role in the nucleolus, where they likely maintain and restore nucleolar organization and integrity during stress (Banski, Kodiha & Stochaj, 2010; Banski, Kodiha & Stochaj, 2011; Kodiha et al., 2005; Kodiha & Stochaj, 2013; Pelham, 1984). Some co-chaperones, such as hsp40 (Hattori et al., 1993, reviewed in Banski, Kodiha & Stochaj, 2011; Kodiha, Frohlich & Stochaj, 2012) are also present in the nucleoli of stressed cells and could thus modulate chaperone functions (Banski, Kodiha & Stochaj, 2011). The data presented here indicate a different role for HspBP1, as the co-chaperone will likely have little impact on nucleolar-specific hsc70 activities during heat shock and recovery.

### Colocalization of hsc70 and HspBP1 in control and stressed cells

To further examine the interplay between hsc70 and HspBP1, we quantified their colocalization at the single-cell level and under different conditions. This goes beyond the measurements in Figs. 1, 3 and 4, as we focused on the *area of overlap* in nuclear and cytoplasmic compartments. To this end, we determined the colocalization for individual pixels, with a pixel size of 0.14 µm^2^.

**Figure 7:**
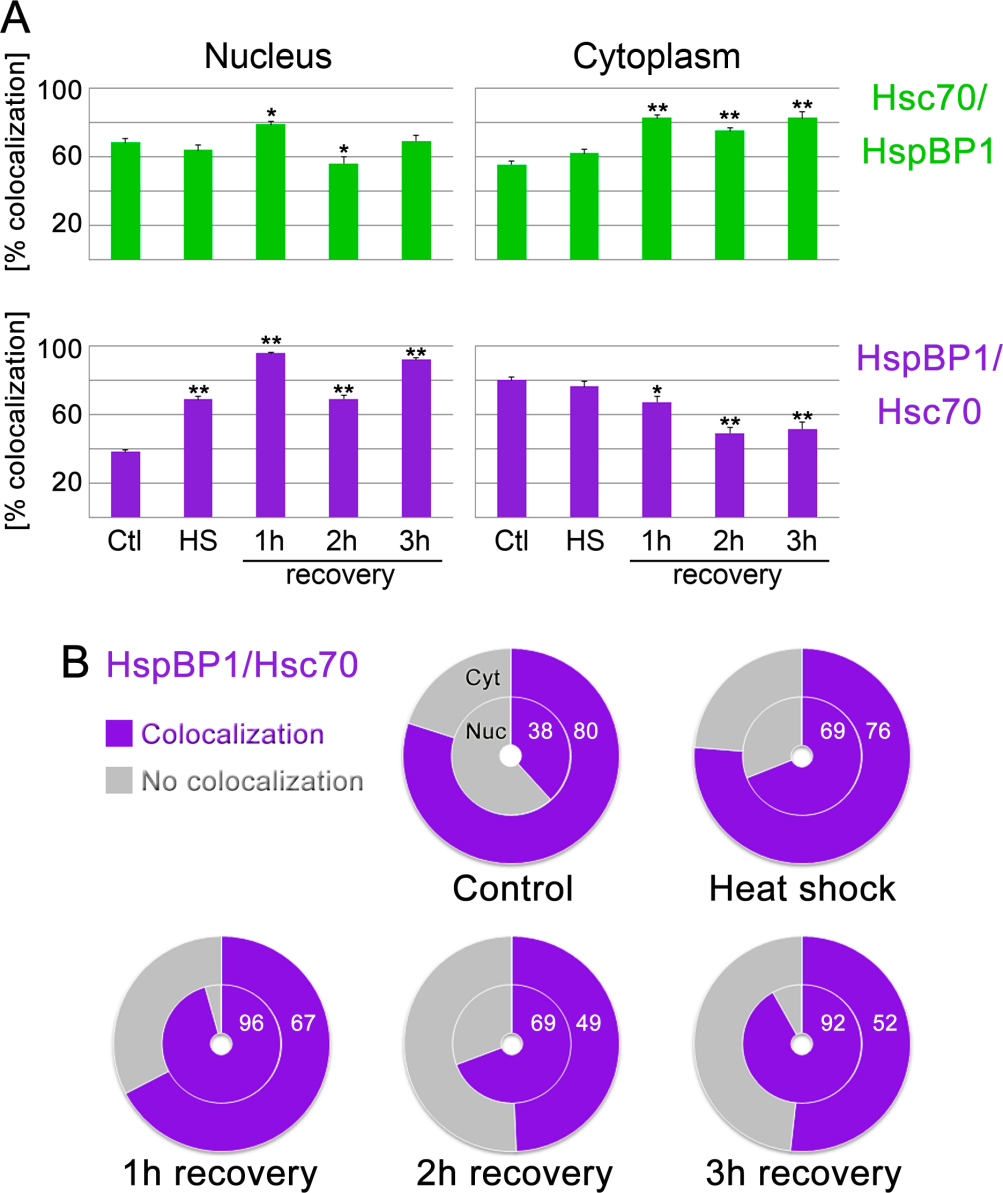
Colocalization of HspBP1 and hsc70 in the nucleus and cytoplasm. (A) Areas of overlapping distribution were identified for Hsc70/HspBP1 and HspBP1/Hsc70 as described in Figure S2. A minimum of ten different cells was analyzed for each data point. Significant differences were identified by One-way ANOVA combined with Bonferroni post-hoc analysis. Pairwise comparisons were performed with control samples as reference; ^∗^, *p* < 0.05; ^∗∗^, *p* < 0.01. (B) Changes in the HspBP1/Hsc70 colocalization are shown both for the cytoplasm (outer ring of the pie graph) and nucleus (inner ring) for all conditions. In pixels containing HspBP1, hsc70 was present (colocalization, purple) or absent (no colocalization, grey). Numbers denote the percentage of HspBP1/Hsc70 colocalization.

Two different aspects of the chaperone/co-chaperone colocalization were assessed. First, the colocalization of hsc70 with HspBP1 was quantified (Fig. 7A; Hsc70/HspBP1, green bars); second, the colocalization of HspBP1 with hsc70 was measured (Figs. 7A and 7B; HspBP1/Hsc70; purple bars and pie charts).

The most significant changes for hsc70/HspBP1 occurred in the cytoplasm during stress recovery. The colocalization increased from approximately 60% to 80%; this means that during recovery ∼80% of the area occupied by hsc70 also contained HspBP1. One possible interpretation is the retention of hsc70 in the cytoplasm through interaction with HspBP1, whereas hsc70 which does not co-localize with HspBP1 moves to the nucleus.

For HspBP1, the changes in colocalization were particularly profound in the nucleus. In unstressed cells, 38% of nuclear HspBP1 colocalized with hsc70 (Fig. 7). Nuclear colocalization increased significantly during and after heat shock, rising to >65%. By contrast, the colocalization of HspBP1 with hsc70 in the cytoplasm was high in control and heat-stressed cells, but diminished during recovery (Fig. 7; from 80% to ∼50%).

In summary, our data indicate that the interplay between HspBP1 and hsc70 is dictated by the availability of each of the two proteins in the nuclear and cytoplasmic pools. Stressed cells can quickly accomplish this by increasing the local protein concentration (for instance, cytoplasmic HspBP1, Fig. 3) or by promoting nucleocytoplasmic relocation (hsc70, Fig. 1). Thus, the regulation of chaperone/co-chaperone function not only depends on their absolute cellular concentrations, but also on their subcellular distribution and colocalization within the compartment.

## Conclusions

Our current study provides a quantitative analysis of the abundance and subcellular distribution of the chaperone/co-chaperone pair hsc70/HspBP1. In addition, we assessed compartment-specific changes in protein localization and identified possible links between hsc70 and HspBP1. Our work goes beyond the measurements of stress-dependent changes in hsc70 and HspBP1 distribution, as we quantified the impact of hyperthermia on hsc70/HspBP1 colocalization. To our knowledge, quantitative in-depth spatial information was previously not available for HspBP1. To obtain these insights, human cancer cells were examined under normal, heat shock and recovery conditions.

We demonstrate a stress-dependent relocation of hsc70; this redistribution is not shared by HspBP1. Interestingly, single-cell analyses uncovered that stress has distinct consequences for hsc70/HspBP1 in the nucleus and cytoplasm. Based on our results, we propose that the subcellular localization and compartment-dependent colocalization control the cooperation of this chaperone/co-chaperone pair.

Heat shock shifts the location of hsc70 towards the nucleus in stressed cells (Figs. 1, 3 and 4) and to nucleoli during recovery (Fig. 6). By contrast, no significant global redistribution was observed for HspBP1. Hsc70 relocation and sequestration (Kodiha et al., 2005) will restrict hsc70/HspBP1 interactions in a compartment-specific fashion and could have important physiological consequences. For instance, a HspBP1:chaperone ratio of 4:1 is required to inhibit 50% of the chaperone function (Raynes et al., 2003). The stress-induced nuclear and nucleolar relocation of hsc70 could thus strengthen the inhibitory role of HspBP1 in the cytoplasm. At the same time, liberation from hsc70 may promote other functions of HspBP1 that are unrelated to its chaperone partner. Possible examples of such processes include the inhibition of the ubiquitin ligase CHIP and the stabilization of specific CHIP targets (Alberti et al., 2004; Rogon et al., 2014).

Alterations in the hsc70/HspBP1 interplay will also affect hsc70 performance, because co-chaperones can modulate the interaction with clients. For example, when compared to Bag-1M, HspBP1 diminishes hsc70 binding to steroid receptors (Knapp et al., 2014). Therefore, it is conceivable that changes in the hsc70/HspBP1 colocalization during and after hyperthermia shift the hsc70 client profile.

While our study focuses on HspBP1, a comprehensive analysis of localized hsc70 functions in the future will also incorporate other co-chaperones, including those co-factors that differ in their mode of action (Bracher & Verghese, 2015; Tzankov et al., 2008). It is noteworthy in this context that co-chaperones vary in their response to stress, which can alter their abundance and localization. For example, heat shock impinges on Bag1, a major hsc70 nucleotide exchange factor (Townsend et al., 2003). Bag1 localization is sensitive to hyperthermia and other stressors; this in turn regulates Bag1’s function, which is determined by its subcellular localization (Liman et al., 2008; Townsend et al., 2003). Another co-chaperone that relocates upon stress is Bag3. The protein accumulates in nuclei upon heat shock, where it modulates the heat shock response through Hsf-1 (Jin, Ahn & Kim, 2015).

Subcellular compartments differ in their ability to cope with stress, and the nucleus is especially sensitive to heat shock (Hageman et al., 2007). As shown by us, heat shock and stress recovery alter the distribution of hsc70, with less pronounced effects on HspBP1. This may reflect compartment-specific repair activities that are required to restore proteostasis. In an alternative model, HspBP1 has distinct biological functions in the nucleus and cytoplasm, and these compartment-specific functions have to be maintained during stress. Future experiments will have to determine whether and how the subcellular localization defines HspBP1 activities.

Aside from hsc70, HspBP1 also interacts with inducible members of the hsp70 family, whose abundance will increase when cells recover from stress (for example Hattori et al., 1993). HeLa cells produce hsp70s even in the absence of stress (Boisvert et al., 2012). Accordingly, a fraction of HspBP1 interacts with hsp70s during the conditions analyzed by us. Similar to hsc70, hsp70s concentrate in nuclei and nucleoli upon hyperthermia (Haddad & Paulin-Levasseur, 2008; Hattori et al., 1993; Pelham, 1984). Therefore, stress-induced changes in the hsp70/HspBP1 relationship may resemble those described here for hsc70/HspBP1.

The subcellular abundance and localization of heat shock proteins are crucial to cellular proteostasis networks. Posttranslational modifications of network hubs, such as hsp70s, are a possible mechanism to control the network organization (Palotai, Szalay & Csermely, 2008). In particular, heat-induced phosphorylation could reorganize chaperone-networks in stressed cells (Palotai, Szalay & Csermely, 2008). While phosphorylation has little impact on hsc70 turnover in unstressed cells (Ahmad et al., 2012; reviewed in Kodiha, Frohlich & Stochaj, 2012), it is conceivable that posttranslational modifications regulate the hsc70 interaction with co-factors, such as HspBP1.

For simplicity only, we restrict our discussion to modifications within the hsc70/HspBP1 interaction sites. Specifically, hsc70 phosphorylation of Thr265, Thr273 and Ser277 occur in the region that binds HspBP1 (Table 1). Interestingly, Thr265 plays a role in hsc70 nucleolar accumulation (Banski et al., 2010), but it is not known whether co-chaperone association modulates hsc70 targeting to nucleoli. HspBP1 contains multiple posttranslational modifications in the major and minor hsc70 interaction sites. HspBP1 phosphorylation on Ser354 and Ser359 is cell cycle-dependent (Dephoure et al., 2008), but little is known about the physiological relevance of HspBP1 modifications. How posttranslational modifications impact hsc70/HspBP1 binding during different growth conditions is an interesting question to be addressed in future studies.

Taken together, our data show differences in the stress-induced redistribution of proteostasis network components. One possible outcome is the liberation of co-chaperones for new activities.

We propose that compartment-specific changes in hsc70/co-chaperone interactions have important consequences for overall and localized network functions.

## Supplemental Information

10.7717/peerj.1530/supp-1 Supplemental Information 1 Raw data for Fig. 1B

10.7717/peerj.1530/supp-2 Supplemental Information 2 Raw data for Figure 3

10.7717/peerj.1530/supp-3 Supplemental Information 3 Raw Data for Figure 4

10.7717/peerj.1530/supp-4 Supplemental Information 4 Figure S1. Identification of nuclear and nucleolar compartments Crm1 and DAPI provide markers for the nucleus (Kodiha, Brown & Stochaj, 2008). Dark holes in the DAPI staining were used to demarcate nucleoli (Kodiha, Banski & Stochaj, 2011). The software defined nucleolar segments; the segments were overlayed with DAPI, HspBP1 or hsc70 images in the bottom panels.

10.7717/peerj.1530/supp-5 Supplemental Information 5 Figure S2. Measurement of colocalization in the nucleus and cytoplasm Regions of interest (ROI) for individual cells were selected for HspBP1 (purple) and hsc70 (green). Images obtained after thresholding (orange) were used to measure the areas of overlap. Representative results are included to illustrate the colocalization between hsc70 (Image A in data panel) and HspBP1 (Image B).

## Additional Information and Declarations

### Data Availability

The following information was supplied regarding data availability: The research in this article did not generate any raw data.

## References

[ref-1] Ahmad Y, Boisvert FM, Lundberg E, Uhlen M, Lamond AI (2012). Systematic analysis of protein pools, isoforms, and modifications affecting turnover and subcellular localization. Molecular and Cellular Proteomics.

[ref-2] Alberti S, Böhse K, Arndt V, Schmitz A, Höhfeld J (2004). The cochaperone HspBP1 inhibits the CHIP ubiquitin ligase and stimulates the maturation of the cystic fibrosis transmembrane conductance regulator. Molecular Biology of the Cell.

[ref-3] Banski P, Kodiha M, Stochaj U (2010). Chaperones and multitasking proteins in the nucleolus: networking together for survival?. Trends in Biochemical Sciences.

[ref-4] Banski P, Kodiha M, Stochaj U (2011). Exploring the nucleolar proteome: novel concepts for chaperone trafficking and function. Current Proteomics.

[ref-5] Banski P, Mahboubi H, Kodiha M, Shrivastava S, Kanagaratham C, Stochaj U (2010). Nucleolar targeting of the chaperone hsc70 is regulated by stress, cell signaling, and a composite targeting signal which is controlled by autoinhibition. Journal of Biological Chemistry.

[ref-6] Boisvert FM, Ahmad Y, Gierlinski M, Charriere F, Lamont D, Scott M, Barton G, Lamond AI (2012). A quantitative spatial proteomics analysis of proteome turnover in human cells. Molecular and Cellular Proteomics.

[ref-7] Bracher A, Verghese J (2015). GrpE, Hsp110/Grp170, HspBP1/Sil1 and BAG domain proteins: nucleotide exchange factors for Hsp70 molecular chaperones. Sub-Cellular Biochemistry.

[ref-9] Brandvold KR, Morimoto RI (2015). The chemical biology of molecular chaperones—implications for modulation of proteostasis. Journal of Molecular Biology.

[ref-10] Brehme M, Voisine C, Rolland T, Wachi S, Soper James H, Zhu Y, Orton K, Villella A, Garza D, Vidal M, Ge H, Morimoto Richard I (2014). A chaperome subnetwork safeguards proteostasis in aging and neurodegenerative disease. Cell Reports.

[ref-11] Chatr-Aryamontri A, Breitkreutz BJ, Oughtred R, Boucher L, Heinicke S, Chen D, Stark C, Breitkreutz A, Kolas N, O’Donnell L, Reguly T, Nixon J, Ramage L, Winter A, Sellam A, Chang C, Hirschman J, Theesfeld C, Rust J, Livstone MS, Dolinski K, Tyers M (2015). The BioGRID interaction database: 2015 update. Nucleic Acids Research.

[ref-12] Csermely P (2001). Chaperone overload is a possible contributor to ‘civilization diseases’. Trends in Genetics.

[ref-13] Daugaard M, Rohde M, Jäättelä M (2007). The heat shock protein 70 family: highly homologous proteins with overlapping and distinct functions. FEBS Letters.

[ref-14] Dephoure N, Zhou C, Villen J, Beausoleil SA, Bakalarski CE, Elledge SJ, Gygi SP (2008). A quantitative atlas of mitotic phosphorylation. Proceedings of the National Academy of Sciences of the United States of America.

[ref-15] Escusa-Toret S, Vonk WI, Frydman J (2013). Spatial sequestration of misfolded proteins by a dynamic chaperone pathway enhances cellular fitness during stress. Nature Cell Biology.

[ref-16] Finka A, Mattoo RH, Goloubinoff P (2011). Meta-analysis of heat- and chemically upregulated chaperone genes in plant and human cells. Cell Stress and Chaperones.

[ref-17] Finka A, Sood V, Quadroni M, Rios Pde L, Goloubinoff P (2015). Quantitative proteomics of heat-treated human cells show an across-the-board mild depletion of housekeeping proteins to massively accumulate few HSPs. Cell Stress & Chaperones.

[ref-18] Graner MW, Raynes DA, Bigner DD, Guerriero V (2009). Heat shock protein 70-binding protein 1 is highly expressed in high-grade gliomas, interacts with multiple heat shock protein 70 family members, and specifically binds brain tumor cell surfaces. Cancer Science.

[ref-19] Gyurko DM, Soti C, Stetak A, Csermely P (2014). System level mechanisms of adaptation, learning, memory formation and evolvability: the role of chaperone and other networks. Current Protein and Peptide Science.

[ref-20] Haddad N, Paulin-Levasseur M (2008). Effects of heat shock on the distribution and expression levels of nuclear proteins in HeLa S3 cells. Journal of Cellular Biochemistry.

[ref-21] Hageman J, Vos MJ, Van Waarde MAWH, Kampinga HH (2007). Comparison of intra-organellar chaperone capacity for dealing with stress-induced protein unfolding. Journal of Biological Chemistry.

[ref-22] Hartl FU, Bracher A, Hayer-Hartl M (2011). Molecular chaperones in protein folding and proteostasis. Nature.

[ref-23] Hattori H, Kaneda T, Lokeshwar B, Laszlo A, Ohtsuka K (1993). A stress-inducible 40 kDa protein (hsp40): purification by modified two-dimensional gel electrophoresis and co-localization with hsc70(p73) in heat-shocked HeLa cells. Journal of Cell Science.

[ref-24] Hornbeck PV, Zhang B, Murray B, Kornhauser JM, Latham V, Skrzypek E (2015). PhosphoSitePlus, 2014: mutations, PTMs and recalibrations. Nucleic Acids Research.

[ref-25] Jin YH, Ahn SG, Kim SA (2015). BAG3 affects the nucleocytoplasmic shuttling of HSF1 upon heat stress. Biochemical and Biophysical Research Communications.

[ref-26] Kabani M, McLellan C, Raynes DA, Guerriero V, Brodsky JL (2002). HspBP1, a homologue of the yeast Fes1 and Sls1 proteins, is an Hsc70 nucleotide exchange factor. FEBS Letters.

[ref-27] Kakkar V, Meister-Broekema M, Minoia M, Carra S, Kampinga HH (2014). Barcoding heat shock proteins to human diseases: looking beyond the heat shock response. Disease Models & Mechanisms.

[ref-28] Knapp RT, Wong MJH, Kollmannsberger LK, Gassen NC, Kretzschmar A, Zschocke J, Hafner K, Young JC, Rein T (2014). Hsp70 Cochaperones HspBP1 and BAG-1M differentially regulate steroid hormone receptor function. PLoS ONE.

[ref-29] Kodiha M, Banski P, Stochaj U (2011). Computer-based fluorescence quantification: a novel approach to study nucleolar biology. BMC Cell Biology.

[ref-30] Kodiha M, Brown CM, Stochaj U (2008). Analysis of signaling events by combining high-throughput screening technology with computer-based image analysis. Science Signaling.

[ref-31] Kodiha M, Chu A, Lazrak O, Stochaj U (2005). Stress inhibits nucleocytoplasmic shuttling of heat shock protein hsc70. American Journal of Physiology—Cell Physiology.

[ref-32] Kodiha M, Frohlich M, Stochaj U (2012). Spatial proteomics sheds light on the biology of nucleolar chaperones. Current Proteomics.

[ref-33] Kodiha M, Mahboubi H, Stochaj U (2014). Exploring subcellular organization and function with quantitative fluorescence microscopy. Microscopy: advances in scientific research and education.

[ref-34] Kodiha M, Pié B, Wang YM, Flamant E, Boppana NB, Young JC, Separovic D, Cooper E, Stochaj U (2015). Detecting changes in the mitochondrial membrane potential by quantitative fluorescence microscopy. Protocol Exchange.

[ref-35] Kodiha M, Salimi A, Wang YM, Stochaj U (2014). Pharmacological AMP kinase activators target the nucleolar organization and control cell proliferation. PLoS ONE.

[ref-36] Kodiha M, Stochaj U (2013). Chaperones and multitasking proteins in the nucleolus. Proteins of the Nucleolus.

[ref-37] Liman J, Faida L, Dohm CP, Reed JC, Bähr M, Kermer P (2008). Subcellular distribution affects BAG1 function. Brain Research.

[ref-38] Liu X, Fagotto F (2011). A method to separate nuclear, cytosolic, and membrane-associated signaling molecules in cultured cells. Science Signaling.

[ref-39] Mahboubi H, Barisé R, Stochaj U (2015). 5′-AMP-activated protein kinase alpha regulates stress granule biogenesis. Biochimica Biophysica Acta—Molecular Cell Research.

[ref-40] Morimoto RI (2011). The heat shock response: systems biology of proteotoxic stress in aging and disease. Cold Spring Harbor Symposia on Quantitative Biology.

[ref-41] Palotai R, Szalay MS, Csermely P (2008). Chaperones as integrators of cellular networks: changes of cellular integrity in stress and diseases. IUBMB Life.

[ref-42] Pelham HR (1984). Hsp70 accelerates the recovery of nucleolar morphology after heat shock. EMBO Journal.

[ref-43] Pratt WB, Gestwicki JE, Osawa Y, Lieberman AP (2015). Targeting Hsp90/Hsp70-based protein quality control for treatment of adult onset neurodegenerative diseases. Annual Review of Pharmacology and Toxicology.

[ref-44] Raynes DA, Graner MW, Bagatell R, McLellan C, Guerriero V (2003). Increased expression of the Hsp70 cochaperone HspBP1 in tumors. Tumour Biology.

[ref-45] Richter K, Haslbeck M, Buchner J (2010). The heat shock response: life on the verge of death. Molecular Cell.

[ref-46] Rogon C, Ulbricht A, Hesse M, Alberti S, Vijayaraj P, Best D, Adams IR, Magin TM, Fleischmann BK, Höhfeld J (2014). HSP70-binding protein HSPBP1 regulates chaperone expression at a posttranslational level and is essential for spermatogenesis. Molecular Biology of the Cell.

[ref-47] Scheibel T, Buchner J, Starke K, Gaestel M (2006). Protein aggregation as a cause for disease. Molecular chaperones in health and disease.

[ref-48] Shiber A, Ravid T (2014). Chaperoning proteins for destruction: diverse roles of Hsp70 chaperones and their co-chaperones in targeting misfolded proteins to the proteasome. Biomolecules.

[ref-49] Shomura Y, Dragovic Z, Chang HC, Tzvetkov N, Young JC, Brodsky JL, Guerriero V, Hartl FU, Bracher A (2005). Regulation of Hsp70 function by HspBP1: structural analysis reveals an alternate mechanism for Hsp70 nucleotide exchange. Molecular Cell.

[ref-50] Sonna LA, Fujita J, Gaffin SL, Lilly CM (2002). Invited review: effects of heat and cold stress on mammalian gene expression. Journal of Applied Physiology.

[ref-51] Souza A, Albuquerque C, Torronteguy C, Frasson A, Maito F, Pereira L, Duval da Silva V, Zerwes F, Raynes D, Guerriero V, Bonorino C (2009). HspBP1 levels are elevated in breast tumor tissue and inversely related to tumor aggressiveness. Cell Stress and Chaperones.

[ref-52] Stolz A, Wolf DH (2010). Endoplasmic reticulum associated protein degradation: a chaperone assisted journey to hell. Biochimica et Biophysica Acta (BBA)—Molecular Cell Research.

[ref-53] Taipale M, Tucker G, Peng J, Krykbaeva I, Lin ZY, Larsen B, Choi H, Berger B, Gingras AC, Lindquist S (2014). A quantitative chaperone interaction network reveals the architecture of cellular protein homeostasis pathways. Cell.

[ref-54] Tanimura S, Hirano AI, Hashizume J, Yasunaga M, Kawabata T, Ozaki K, Kohno M (2007). Anticancer drugs up-regulate HspBP1 and thereby antagonize the prosurvival function of Hsp70 in tumor cells. Journal of Biological Chemistry.

[ref-55] Townsend PA, Cutress RI, Sharp A, Brimmell M, Packham G (2003). BAG-1 prevents stress-induced long-term growth inhibition in breast cancer cells via a chaperone-dependent pathway. Cancer Research.

[ref-56] Tutar Y, Song Y, Masison DC (2006). Primate chaperones Hsc70 (constitutive) and Hsp70 (induced) differ functionally in supporting growth and prion propagation in Saccharomyces cerevisiae. Genetics.

[ref-57] Tzankov S, Wong MJ, Shi K, Nassif C, Young JC (2008). Functional divergence between co-chaperones of Hsc70. Journal of Biological Chemistry.

[ref-58] Vihervaara A, Sistonen L (2014). HSF1 at a glance. Journal of Cell Science.

[ref-59] Wang XY, Facciponte JG, Subjeck JR, Starke K, Gaestel M (2006). Molecular chaperones and cancer immunotherapy. Molecular chaperones in health and disease.

[ref-60] Yang Z, Zhuang L, Szatmary P, Wen L, Sun H, Lu Y, Xu Q, Chen X (2015). Upregulation of heat shock proteins (HSPA12A, HSP90B1, HSPA4, HSPA5 and HSPA6) in tumour tissues is associated with poor outcomes from HBV-related early-stage hepatocellular carcinoma. International Journal of Medical Sciences.

[ref-61] Young JC, Barral JM, Ulrich Hartl F (2003). More than folding: localized functions of cytosolic chaperones. Trends in Biochemical Sciences.

