# Supplementary figures and images for: Quantitative analysis of the interplay between hsc70 and its co-chaperone HspBP1
Source: PeerJ. 2015 Dec 21;3:e1530. doi: 10.7717/peerj.1530 (PMC4690350; doi:10.7717/peerj.1530)

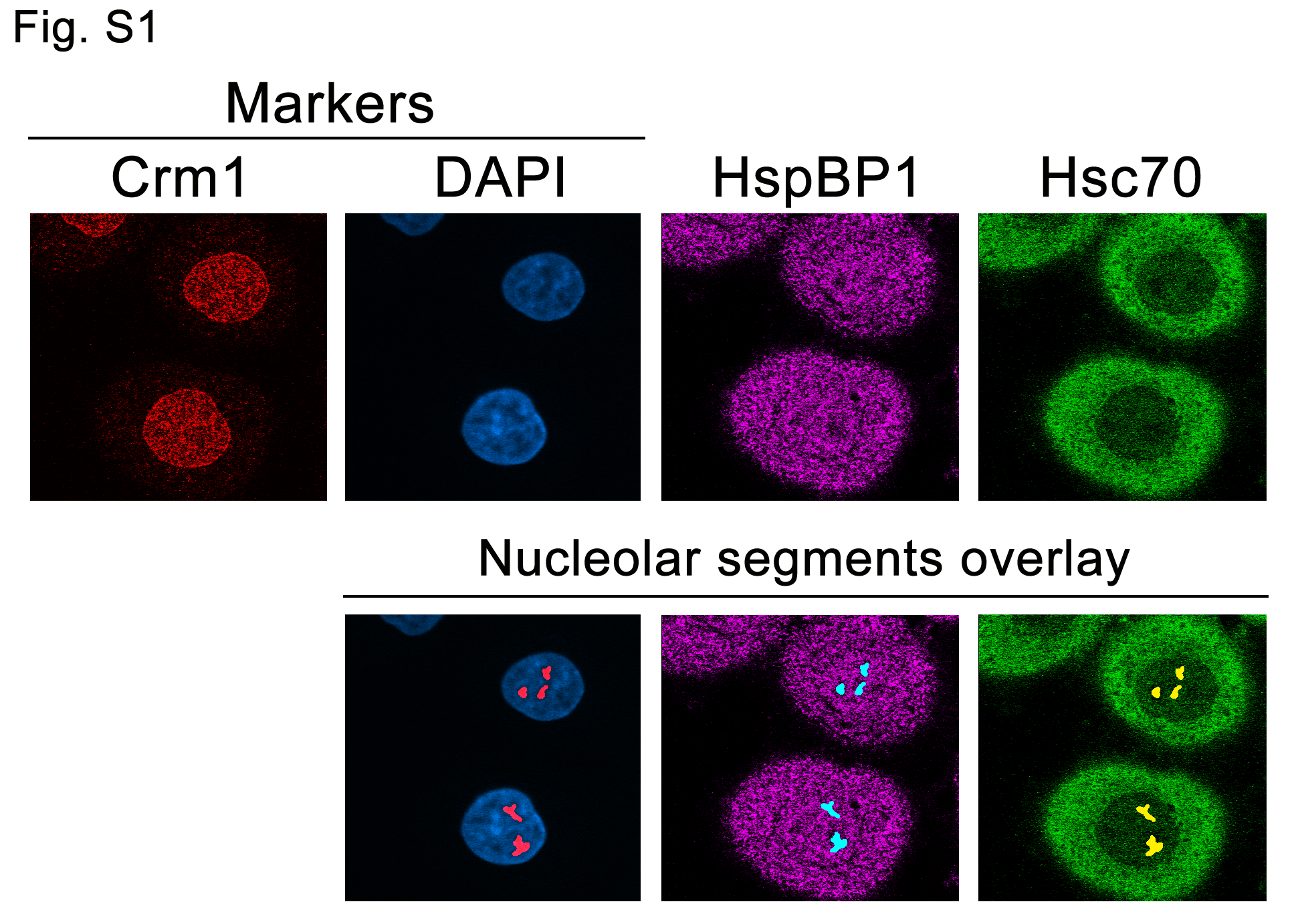

Supplement: Supplemental Information 4 — Crm1 and DAPI provide markers for the nucleus (Kodiha, Brown & Stochaj, 2008). Dark holes in the DAPI staining were used to demarcate nucleoli (Kodiha, Banski & Stochaj, 2011). The software defined nucleolar segments; the segments were overlayed with DAPI, HspBP1 or hsc70 images in the bottom panels. [file peerj-03-1530-s004.png]

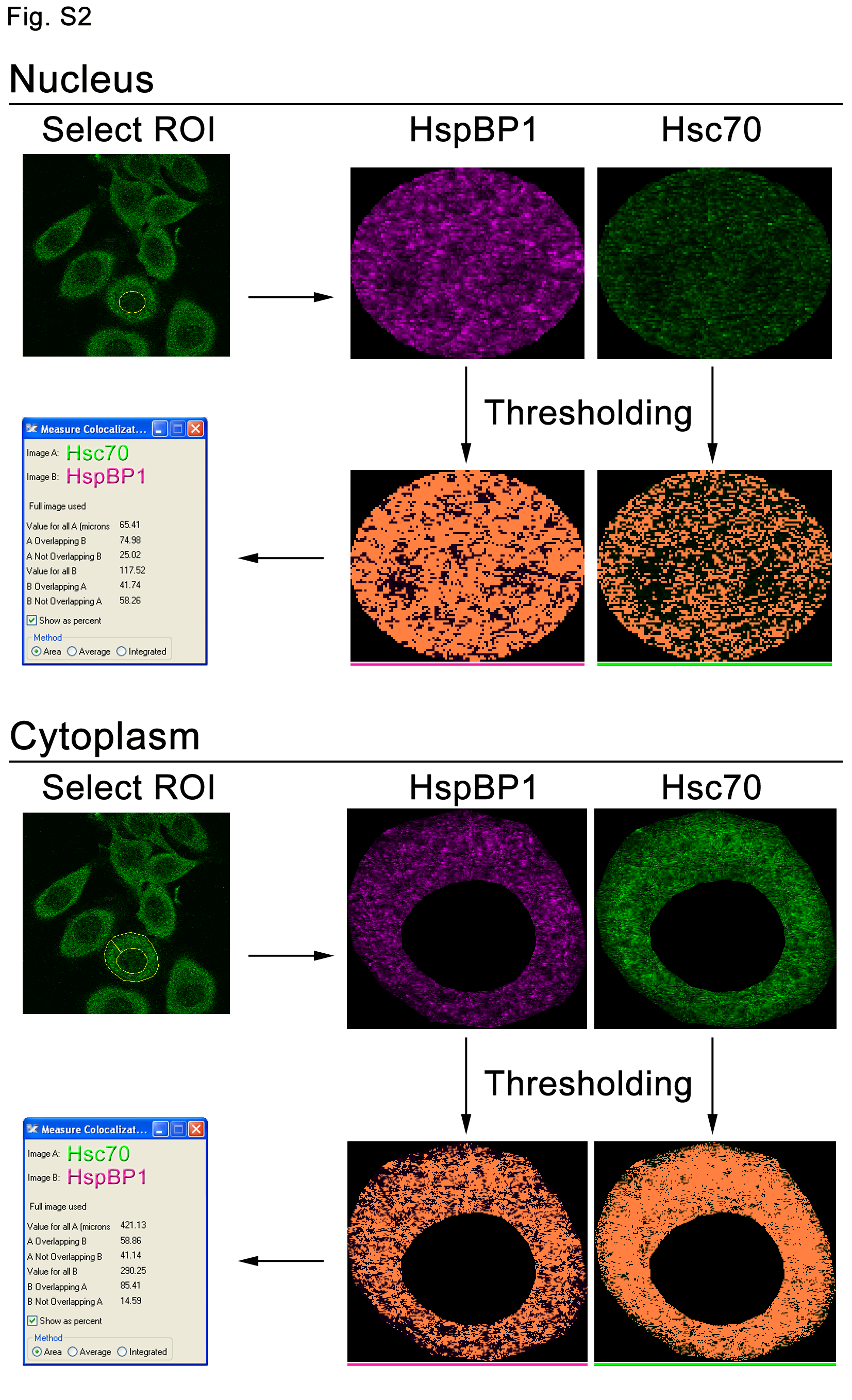

Supplement: Supplemental Information 5 — Regions of interest (ROI) for individual cells were selected for HspBP1 (purple) and hsc70 (green). Images obtained after thresholding (orange) were used to measure the areas of overlap. Representative results are included to illustrate the colocalization between hsc70 (Image A in data panel) and HspBP1 (Image B). [file peerj-03-1530-s005.png]
